# A Novel Co-Crystal Structure Affords the Design of Gain-of-Function Lentiviral Integrase Mutants in the Presence of Modified PSIP1/LEDGF/p75

**DOI:** 10.1371/journal.ppat.1000259

**Published:** 2009-01-09

**Authors:** Stephen Hare, Ming-Chieh Shun, Saumya Shree Gupta, Eugene Valkov, Alan Engelman, Peter Cherepanov

**Affiliations:** 1 Division of Medicine, Imperial College London, St. Mary's Campus, London, United Kingdom; 2 Department of Cancer Immunology and AIDS, Dana-Farber Cancer Institute, Boston, Massachusetts, United States of America; University of Pennsylvania School of Medicine, United States of America

## Abstract

Lens epithelium derived growth factor (LEDGF), also known as PC4 and SFRS1 interacting protein 1 (PSIP1) and transcriptional co-activator p75, is the cellular binding partner of lentiviral integrase (IN) proteins. LEDGF accounts for the characteristic propensity of *Lentivirus* to integrate within active transcription units and is required for efficient viral replication. We now present a crystal structure containing the N-terminal and catalytic core domains (NTD and CCD) of HIV-2 IN in complex with the IN binding domain (IBD) of LEDGF. The structure extends the known IN–LEDGF interface, elucidating primarily charge–charge interactions between the NTD of IN and the IBD. A constellation of acidic residues on the NTD is characteristic of lentiviral INs, and mutations of the positively charged residues on the IBD severely affect interaction with all lentiviral INs tested. We show that the novel NTD–IBD contacts are critical for stimulation of concerted lentiviral DNA integration by LEDGF *in vitro* and for its function during the early steps of HIV-1 replication. Furthermore, the new structural details enabled us to engineer a mutant of HIV-1 IN that primarily functions only when presented with a complementary LEDGF mutant. These findings provide structural basis for the high affinity lentiviral IN–LEDGF interaction and pave the way for development of LEDGF-based targeting technologies for gene therapy.

## Author Summary

Retroviruses crucially rely on insertion of their genomes into a host cell chromosome, and this process is carried out by the viral enzyme integrase. HIV and other lentiviruses also depend on LEDGF, a cellular chromatin-associated protein, which binds their integrase proteins and tethers them to a human chromosome. The interaction between integrase and LEDGF can potentially be exploited for directing integration of lentiviral vectors in gene therapy applications, as well as for development of antiretroviral drugs. Herein, we present a three-dimensional structure of a protein–protein complex containing a fragment of HIV integrase and the integrase-binding domain of LEDGF. Our structure elucidates the hitherto unknown LEDGF–integrase interface involving the amino terminal portion of the viral enzyme. Using a range of complementary approaches, we further show that these novel protein–protein contacts are essential for the function of LEDGF in HIV integration. The novel structural details will be very useful for the development of HIV inhibitors that target the integrase–LEDGF interaction. Furthermore, they enabled us to design a mutant of HIV integrase that depends on a reverse-engineered mutant of LEDGF, providing an inroad to the design of LEDGF-based lentiviral vector targeting strategies.

## Introduction

Integration of reverse transcribed viral cDNA into the host cell genome is an essential step in the retroviral life cycle. This process is catalyzed by integrase (IN), a virus-derived enzyme, which carries out two separate reactions acting on both cDNA termini (reviewed in [1],[2]). Firstly, 3′-processing takes place in the cytoplasm of the host cell, in which a di- or trinucleotide is hydrolytically removed from each cDNA end, exposing 3′-hydroxyl groups of invariant CA dinucleotides. The enzyme remains attached to both viral cDNA ends within a higher order pre-integration complex (PIC). The PIC is transported into the nucleus and, upon locating a suitable chromatin environment, the second reaction, strand transfer, ensues. During this step, the pair of hydroxyl groups produced during 3′-processing nick and join to opposing strands of the cellular DNA, four to six base pairs apart, depending on the retroviral genus. To complete the process, cellular enzymes repair the integration site, resulting in a stable provirus flanked by short duplications of the target DNA sequence.

Retroviral INs share a conserved three domain organization, each containing a central catalytic core domain (CCD), flanked by N- and C-terminal domains (NTD and CTD) [3]–[5]. The CCD spans the most conserved region of IN and bears close structural homology to prokaryotic transposases [6]. The enzyme active site is comprised of three invariant acidic residues (the D,DX_35_E motif) that coordinate a pair of Mg^2+^ cations during catalysis [7],[8]. The NTD forms a three-helical bundle, which folds around a zinc atom coordinated by His and Cys residues of an HHCC motif [9],[10]. The CTD features an SH3-like fold, is rich in basic residues and is likely involved in DNA binding [11],[12]. Despite Herculean efforts directed towards characterization of this key antiviral drug target, the structure of a full-length retroviral IN remains elusive. The active form of retroviral IN is a tetramer [13]–[15], and a plausible tetramer model for the apoenzyme was proposed based on a crystal structure of a two-domain fragment of HIV-1 IN containing its NTD and CCD (IN_NTD+CCD_) [16].

Lentiviral DNA integration critically depends on lens epithelium-derived growth factor (LEDGF) (reviewed in [17]). LEDGF tightly associates with chromatin and has been implicated in regulation of cellular gene expression, epigenetic chromatin modifications and apoptosis [18]–[20]. The host factor directly binds HIV-1, HIV-2, as well as other lentiviral INs and dramatically stimulates their strand transfer activity [21]–[24]. LEDGF tethers lentiviral IN to host chromatin in the nucleus [24]–[27] and plays a critical role in directing PICs to active genes during integration [28]–[32]. LEDGF contains a pair of small structural domains: an ∼92 residue PWWP domain at its N-terminus, responsible for binding to an as yet unidentified component of chromatin, and the IN binding domain (IBD, residues 347–429) within its C-terminal portion [33]–[35]. The CCD and NTD of IN were both implicated in LEDGF binding: while the CCD is minimally sufficient, the NTD is required for high affinity binding [27],[36]. Deletion of the HIV-1 IN NTD, or a mutation destabilizing zinc coordination within this domain (His-12 to Asn), greatly reduced the interaction with LEDGF [27]. A close homolog of LEDGF, hepatoma derived growth factor-related protein 2 (HRP2), contains conserved PWWP and IBD-like domains. Although HRP2 is able to interact with HIV-1 IN and stimulate its enzymatic activity *in vitro* [33], it remains to be established whether it plays a role in lentiviral integration.

The structure of the LEDGF IBD, composed of a pair of α-helical hairpins, has been determined both separately and in complex with the HIV-1 IN CCD [34],[36]. In the IN_CCD_:LEDGF_IBD_ complex, Ile-365 of LEDGF inserts into a hydrophobic pocket at the IN dimer interface. The interaction is further bolstered by additional hydrophobic interactions between IN residue Trp-131 and LEDGF Phe-406 and Val-408. Asp-366 of LEDGF plays an important role in protein-protein recognition, forming a pair of essential hydrogen bonds with the main chain amides of IN residues Glu-170 and His-171 [36]. Mutation of LEDGF Asp-366 to Asn ablated the interaction with all lentiviral INs tested so far, indicating a common mechanism of recognition [22]. In this work we extend the known lentiviral IN-LEDGF interface to include contacts between the IN NTD and the IBD of LEDGF. This part of the protein-protein interface is essential for high affinity binding and stimulation of concerted DNA integration, and allows designs of complementary pairs of IN and LEDGF mutants for practical uses in gene therapy.

## Results

### Crystallization and Structure Determination

To further characterize the interface between lentiviral INs and LEDGF, we obtained a complex of HIV-2 IN_NTD+CCD_ and LEDGF_IBD_ by co-expression, and crystallized it in two forms. Although crystal form II diffracted to slightly lower resolution than form I (Table 1), it resulted in a higher quality structure. Firstly, form I displayed a higher degree of disorder and only three quarters of the asymmetric unit (ASU) could be unambiguously defined in electron density maps. Secondly, the twelve-fold non-crystallographic symmetry (NCS) in form II dramatically increased the observations∶parameters ratio, resulting in a pseudo-high resolution structure. The structures observed in both crystal forms were overall equivalent, and the remainder of the paper will focus on form II. Snapshots of electron density for the CCD-IBD interface and Zn-His_2_Cys_2_ cluster of the NTD are shown in Figure S1A and S1B. Of note, all previous HIV-1 IN CCD crystal structures required Phe-185 to be mutated to Lys or His to improve protein solubility. Such a mutation was not necessary to crystallize the HIV-2 IN_NTD+CCD_:LEDGF_IBD_ complex. Therefore, our structure is the first to include an HIV IN CCD with a Phe residue naturally occurring at this position (Figure S1C).

**Table 1:** Data collection and refinement statistics.

		Crystal Form I	Crystal Form II
**Data Collection**	Space group	P321	P2_1_2_1_2_1_
	Cell dimension a, b, c (Å)	210.5, 210.5, 162.6	201.4, 202.5, 280.5
	Resolution (Å)	44-3.06 (3.22-3.06)*	50-3.2 (3.37-3.20)
	**Total reflections**	**480,127**	**1,480,594**
	**Unique reflections**	**77,620**	**188,459**
	R_merge_ (%)	8.3 (52.1)	16.1 (56.2)
	I/σ(I)	16.1 (3.0)	13.0 (3.9)
	Completeness (%)	99.1 (99.6)	99.9 (100)
	Redundancy	6.2 (6.0)	7.9 (8.0)
**Refinement:**	Resolution (Å)	35-3.06	35-3.2
	Number of reflections used	73,711	178,727
	R_work_/R_free_(%)	27.3/29.6	22.5/23.4
	Number of atoms: Protein	15652	46692
	Number of atoms: Ligand/ion	48	48
	R.m.s. deviations from ideal bond length (Å)	0.015	0.006
	R.m.s. deviations from ideal bond angles (°)	1.900	0.948
	Ramachandran (%) most favored	87.4	91.4
	Ramachandran (%) Additionally allowed	10.7	8.4
	Ramachandran (%) Generally allowed	1.5	0.2
	Ramachandran (%) Disallowed	0.4	0

*: Values in parentheses are for the highest resolution shell.

**Figure 1:**
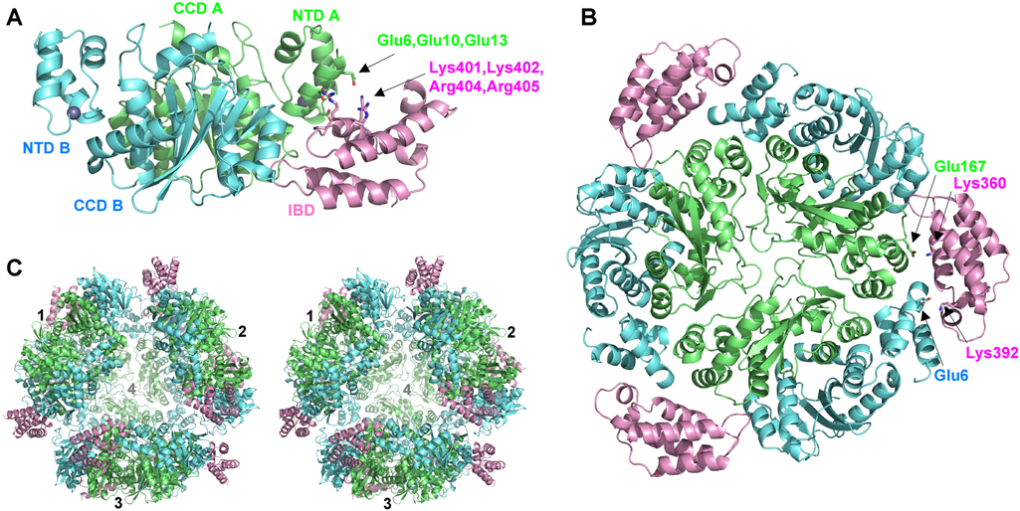
The HIV-2 IN_NTD+CCD_:LEDGF_IBD_ Structure and Multimeric Assemblies Found in the Crystal. (A) The IN_2_LEDGF substructure, containing a dimer of IN_NTD+CCD_ and a single molecule of LEDGF_IBD_, the basic building unit of both crystal forms. IN chains are colored pale green and cyan, and LEDGF is pink. Zinc atoms are shown as gray spheres. Residues involved in IN–LEDGF interfaces and discussed in the text are shown as sticks and indicated with arrowheads. (B) The closed trimer of IN_2_LEDGF substructures. Colors and labels as in (A). (C) Stereo view of the higher order assembly involving four IN_2_LEDGF trimers representing the entire ASU of crystal form II. Colors as in (A).

Based on elution from calibrated gel filtration columns and velocity analytical ultracentrifugation experiments, the purified HIV-2 IN_NTD+CCD_:LEDGF_IBD_ complex behaved as a monodisperse species with a calculated molecular mass of ∼60 kDa (data not shown). This size is consistent with a dimer of HIV-2 IN_NTD+CCD_ plus one or two LEDGF_IBD_ molecules, closely matching the basic building unit observed in the crystal (Figure 1A, referred to as the IN_2_LEDGF substructure). The substructure assembles further into closed trimers with a three-fold NCS (Figure 1B), and four such trimers accrue in the ASU to form a spherical structure containing 24 IN and 12 LEDGF chains (Figure 1C). The trimer is held primarily via IN-IN (CCD-CCD, NTD-CCD and NTD-NTD) interactions (Figure 1B), and the total buried surface area between neighboring IN dimers is ∼2,000 Å^2^.

### The IN–LEDGF Interface and the Role of the IN NTD

A total of ∼1,450 Å^2^ of molecular surface is buried at the IN-LEDGF interface within the IN_2_LEDGF substructure. HIV-1 and HIV-2 INs share ∼60% amino acid sequence identity over the span of their CCDs. Accordingly, the contacts between the HIV-2 IN CCD and the IBD are very similar to those observed in the HIV-1 IN_CCD_:LEDGF_IBD_ structure, and have been extensively discussed elsewhere [36]. Significant changes to this part of the interface occur due to amino acid replacement at positions 128 and 129: HIV-2 encodes Met and Val, respectively, while HIV-1 carries Ala in both cases. The HIV-2 residues are nevertheless involved in similar hydrophobic interactions: the Met-128 side chain packs against Leu-368, Phe-406, and Val-408 of LEDGF, while Val-129 contributes to the hydrophobic pocket that buries LEDGF residue Ile-365 (Figure S2). As predicted [22],[36], the critical LEDGF Asp-366 residue forms a bidentate hydrogen bond to the same backbone amides in HIV-2 and HIV-1 INs, even though the side chains at these positions differ between viruses (Asn-170 and Thr-171 in HIV-2; Glu-170 and His-171 in HIV-1).

**Figure 2:**
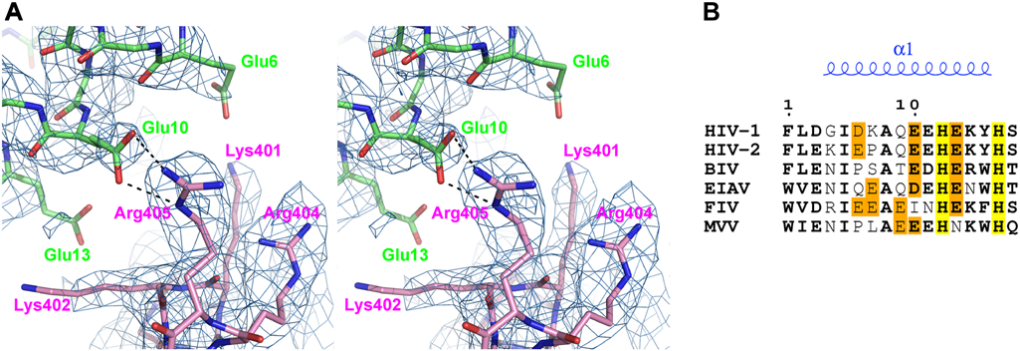
Details of the NTD–IBD Interface. (A) The acidic IN residues and basic LEDGF residues are shown as sticks. The 2Fo-Fc electron density map, contoured at 1σ, is shown in pale blue. (B) Partial amino acid sequence alignment including residues 1–17 of human (HIV-1 and HIV-2) and non-primate (BIV, EIAV, FIV, and MVV) lentiviral INs. Conserved residues are shown in bold; His-12 and His-16 of the invariant HHCC motif are highlighted in yellow. Acidic residues known (for HIV-1 and HIV-2) or proposed to interact with the positively charged face of the IBD are orange.

In agreement with prior biochemical analyses [27], the NTD of IN makes extensive contacts with LEDGF. A constellation of acidic residues on the first helix (α1) of the NTD (Glu-6, Glu-10, and Glu-13) faces positively charged residues on the α4 helix of the IBD (Lys-401, Lys-402, Arg-404, and Arg-405). Side-chains of LEDGF residues Lys-401, Arg-404, and Arg-405 are well ordered, and a well-defined salt bridge involves IN residue Glu-10 and Arg-405 of LEDGF (Figure 2A). The remaining side chains show varying degrees of order and appear to contribute to the overall charges of the interacting faces. The closely positioned and highly conserved IN residue Glu-11 is not involved in the interface and instead interacts with Lys-25 and Lys-186 of the same IN chain, supporting NTD structural integrity and hence overall stability of the IN_2_LEDGF substructure.

Acidic residues at IN positions 6, 10, and 13 are highly conserved among HIV isolates, and those at positions 10 and 13 tend to be acidic within *Lentivirus*, whose members retain at least one of the two negative charges (Figure 2B). Feline immunodeficiency virus (FIV) and maedi-visna virus (MVV) INs that lack negative charges at positions 10 and 13, respectively, contain additional acidic residues (Glu-7 and/or Glu-9), which should preserve the negative charge of the NTD face. Overall, lentiviral INs maintain two or more acidic residues within α1 of their NTDs, which can be predicted to contribute to the interaction with the positive face of the IBD. Conversely, the complementary basic residues are conserved among all known LEDGF and HRP2 orthologs, with some variation only at the position corresponding to LEDGF Arg-405, where Arg or Lys is accommodated [22],[24],[33],[34]. Of note, although some INs from nonlentiviral genera contain acidic residues at positions corresponding to residues 10 or 13 of lentiviral INs, these residues are not conserved within or among these genera (data not shown).

The pair of NTDs belonging to the IN dimer of the IN_2_LEDGF substructure exist in equivalent orientations with respect to the CCD dimer and are supported by contacts with the CCDs involving three salt bridges (Glu-11:Lys-186, Lys-20:Asp-193, and Glu-21:Arg-188) as well as hydrophobic stacking interactions involving Lys-14 and Tyr-15 of the NTD and Trp-131, Trp-132, and Lys-186 of the CCD. An almost identical NTD-CCD interface was observed in the crystal structure of the uncomplexed HIV-1 IN_NTD+CCD_ tetramer [16] (Figure S3B, discussed in more detail below). Notably, the interface was formed between a CCD of one IN dimer (green in Figure S3B) and an NTD from another (yellow in Figure S3B), and so the two structures present an interesting case of domain swapping (Figure S3). The other NTD of the IN_2_LEDGF substructure (cyan in Figure S3A) is important in forming the closed trimers as it interacts with a second IN_2_LEDGF module through its A chain NTD and the IBD (Figure 1B).

### The NTD–IBD Interface Is Critical for the High Affinity IN–LEDGF Interaction and Affords Functional Charge Reversal of Opposing Molecular Faces

**Figure 3:**
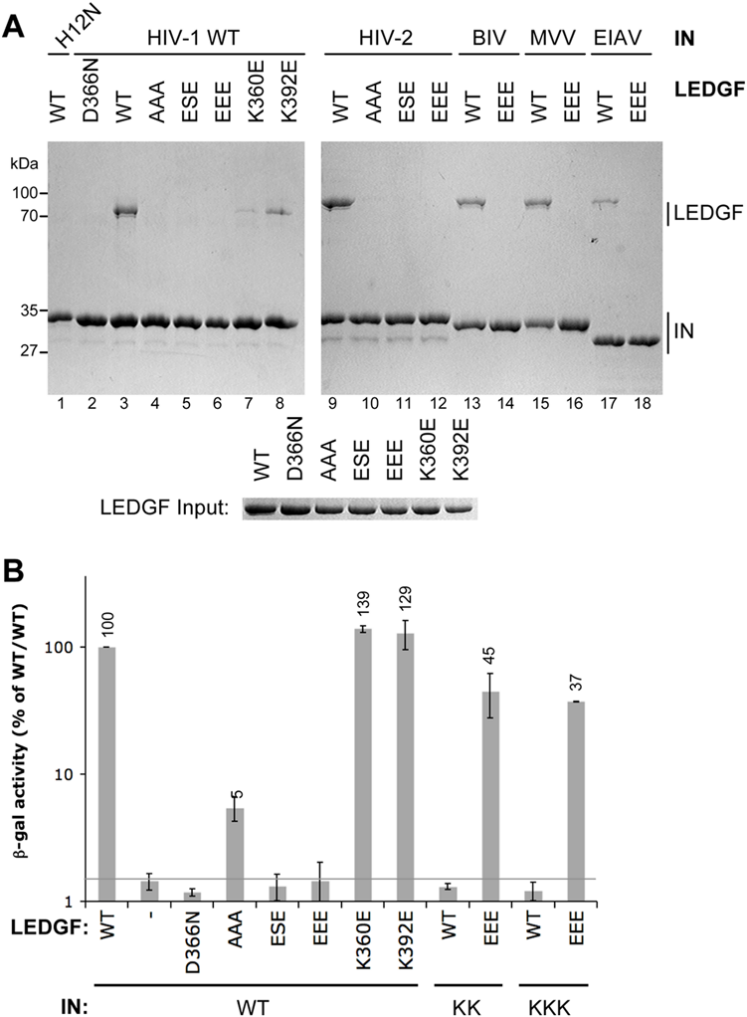
Functional Importance of the NTD–IBD Charge–Charge Interface for the Lentiviral IN–LEDGF Interaction. (A) Pull down experiments. Non-tagged WT, D366N, AAA, ESE, EEE, K360E, or K392E LEDGF proteins were incubated with C-terminally His_6_-tagged forms of HIV-1 IN mutant H12N, WT HIV-1 IN, HIV-2 IN, BIV IN, MVV IN, or EIAV IN (as indicated) in the presence of Ni-NTA agarose. Bound proteins were separated in 11% sodium dodecyl sulfate polyacrylamide gels and visualized with Coomassie Blue. Input quantities of LEDGF proteins are shown below the main gel. (B) Yeast two-hybrid analyses. The IN–LEDGF interaction assay [22],[38] is based on transactivation of the β-galactosidase gene in *S. cerevisiae* indicator cells Y187. Relative β-galactosidase activities produced by yeast co-transformed with the indicated IN and LEDGF_IBD_ mutants are shown on a log scale, with 100% corresponding to the WT bait/WT prey condition. The background of the assay is defined by WT IN in the presence of empty prey vector and is shown as a gray line. Each bar represents a mean value; standard deviations were calculated based on results of quadruplicate measurements. Values exceeding the background are shown atop the bars.

The domain–domain interfaces observed in the crystal structure were targeted by mutagenesis to investigate their functional relevance. Three LEDGF mutants were designed to eliminate or reverse the positive charges facing the IN NTD: K401A/K402A/R405A (AAA), K401E/K402S/R405E (ESE), and K401E/K402E/R405E (EEE). The K360E mutation targeted a salt bridge from LEDGF residue Lys-360 to IN Asp-167 within the IBD-CCD interface [36], and K392E was made to disrupt a potential interaction between Lys-392 and Glu-6 within the secondary NTD-IBD interface that contributes to substructure trimerization (Figure 1B). The mutants were tested in a His_6_-tag pull-down assay for binding to the INs from HIV-1, HIV-2, and three nonprimate lentiviruses (bovine immunodeficiency virus [BIV], MVV, and equine infectious anemia virus [EIAV]). Consistent with earlier reports [22],[27], wild type (WT) LEDGF was pulled down by all WT lentiviral INs, but not with the HIV-1 H12N mutant (Figure 3A). His-12 is involved in zinc coordination and is, therefore, critical for structural integrity of the NTD [37]. Also in agreement with prior work [22],[34], D366N LEDGF did not interact with HIV-1 IN (Figure 3A) or any of the remaining INs (data not shown). Mutations reversing the positively charged face of the IBD disrupted binding to HIV-1 and HIV-2 INs (Figure 3A, lanes 5, 6, 11, and 12), and only very weak binding was detected for AAA LEDGF (lanes 4 and 10). Furthermore, the EEE mutations ablated the interaction with nonprimate lentiviral INs (lanes 14, 16, 18; data not shown). HIV-1 IN binding to LEDGF mutant K360E was also affected, albeit to a lesser degree (lane 7), while K392E was pulled down most efficiently of all LEDGF mutants tested (lane 8).

The yeast two-hybrid technique proved more sensitive than pull down analyses when applied to weak interactions between IN and LEDGF mutants [38]. Full-length HIV-1 IN fused to the DNA binding domain of Gal4 serves as bait, and binding of the LEDGF IBD fused to Gal4 transcription activation domain is reflected by β-galactosidase reporter gene activity. In this assay, LEDGF mutants K360E and K392E showed wild type levels of binding to HIV-1 IN, AAA bound at about 5% of WT, while similarly to D366N, the ESE and EEE mutants failed to interact at detectable levels (Figure 3B), essentially corroborating the results of the His_6_-tag pull down experiments. To demonstrate that these observations were not due to off-site effects such as defective folding or reduced expression of the LEDGF mutants and to validate the novel NTD-IBD interface further, the complementary IN residues were mutated, producing a reversed charge D6K/E10K/E13K (KKK) HIV-1 mutant. Impressively, KKK IN robustly interacted with the complementary EEE LEDGF mutant, generating ∼40% of WT-WT β-galatosidase activity and furthermore, it failed to interact with WT LEDGF (Figure 3B). The double HIV-1 IN E10K/E13K (KK) mutant bearing charge reversals at the two more conserved acidic positions (Figure 2B) likewise gained the ability to interact with EEE LEDGF while displaying no detectable binding to the WT protein in this assay (Figure 3B).

### The NTD–IBD Interface and LEDGF-Dependent HIV-1 IN Strand Transfer Activity

**Figure 4:**
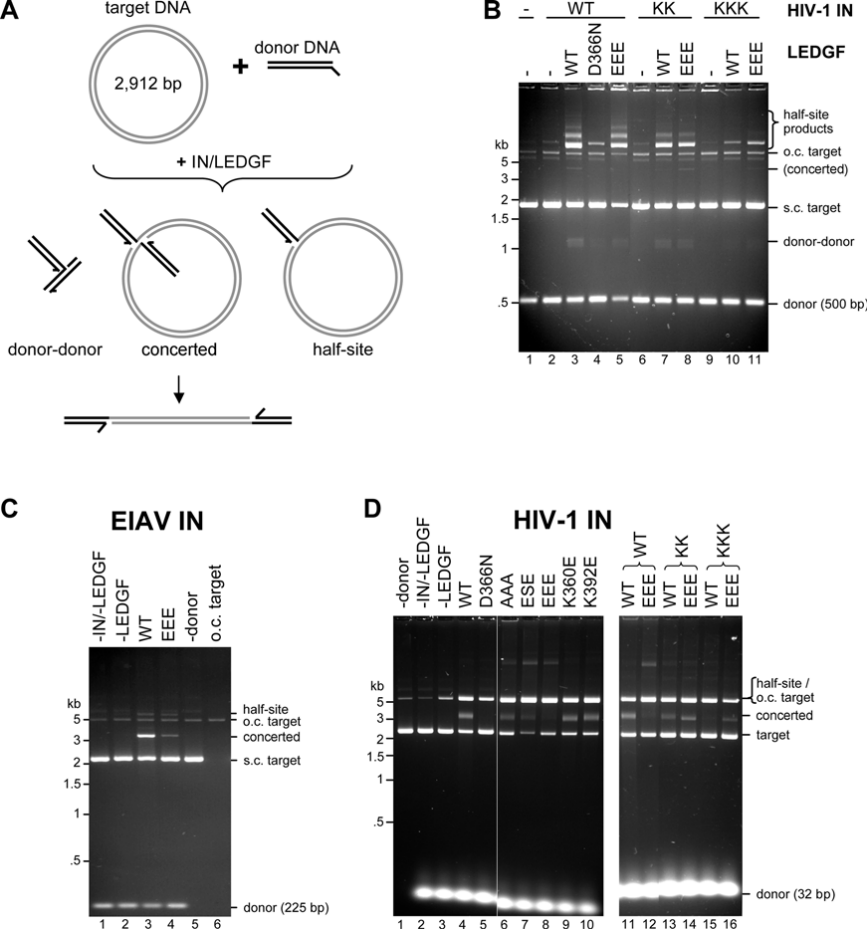
Effects of Mutations within the NTD–IBD Interface on LEDGF–Dependent Strand Transfer Activities of Lentiviral INs. (A) Schematic of the reactions mediated by IN in the presence of circular DNA target: concerted integration results in a linear product while half-site reactions produce branched molecules (circular half-site and donor-donor). (B) HIV-1 IN activity under established conditions that favor half-site integration [22]. WT, KK, or KKK HIV-1 IN was incubated with 10 nM 500-bp donor DNA and supercoiled target DNA in the absence (lane 2) or presence (lanes 3–11) of WT, D366N, or EEE LEDGF. The products separated in an agarose gel were detected with ethidium bromide. Lane 1 contained a mock sample, with both IN and LEDGF omitted. (C) EIAV IN reactions. EIAV IN was incubated with 10 nM 225-bp donor DNA and supercoiled target DNA in the absence (lane 2) or presence (lanes 3 and 4) of WT or EEE LEDGF, as indicated. Lane 1 contained a mock sample without IN and LEDGF; lane 5 contained IN and WT LEDGF without donor DNA; lane 6 contained 10 ng of singly-nicked pGEM-9Zf(-) [22] as a migration standard for the open circular (o.c.) form of target DNA. (D) Enhanced concerted HIV-1 integration assay using an elevated input of donor DNA (for details, see Text S1 and Figure S4). WT, KK, or KKK HIV-1 IN was incubated with 0.5 µM pre-processed 32-bp donor and supercoiled target DNA in the presence of WT, D366N, AAA, ESE, EEE, K360E, or K392E LEDGF. Migration positions of various DNA species (size standards, supercoiled [s.c.] and o.c. target, donor, and reaction products) are indicated.

Whereas *in vivo* retroviral INs must integrate both viral cDNA ends in a concerted fashion, their recombinant forms are typically more proficient at half-site integration (*i.e.* integration of a single viral DNA end into one strand of a target DNA molecule). The relative efficiencies of concerted and half-site integration processes strongly depend on the *in vitro* reaction conditions, with parameters such as the length of the donor DNA, enzyme source and concentration, and presence of crowding agents greatly affecting the outcomes [22], [39]–[41]. The use of a short mimic of the viral cDNA end (referred to as donor DNA substrate) and supercoiled target DNA conveniently allows discrimination between products of concerted and half-site integration (Figure 4A).

LEDGF robustly promotes the strand transfer activities of divergent lentiviral INs *in vitro*, although the fidelity of LEDGF-mediated strand transfer varies for the different INs [21],[22]. Intriguingly, while simulating half-site strand transfer activity of HIV-1 IN under all reported conditions, the host factor has the capacity of to either inhibit [42] or bolster [43] its concerted integration activity.

In the presence of 10 nM 500-bp donor substrate, 2.9 kb supercoiled plasmid DNA (pGEM), and WT LEDGF, WT HIV-1 IN carries out robust, predominantly half-site strand transfer [22] (Figure 4B, lane 3); half-site products migrate in agarose gels well above the open circular form of the target, while concerted integration products appear as linear (∼4,000 bp) DNA species (Figure 4A and 4B). In agreement with earlier observations, the reaction was severely affected by the critical LEDGF D366N mutation (Figure 4B, lane 4). Despite greatly reduced binding affinity, the EEE LEDGF mutant retained the ability to stimulate half-site strand transfer activity, evident from accumulation of both donor-target and donor-donor products (lane 5). Concordantly, both WT and EEE LEDGF proteins stimulated the half-site activity of KK and KKK HIV-1 IN mutants. The KKK mutant, while significantly less active than WT IN, was somewhat more responsive to the mutant form of LEDGF (compare lanes 10 and 11). Based on these observations we conclude that the intact NTD-IBD interface and hence the full affinity of the IN-LEDGF interaction is not required for stimulation of half-site integration. This finding was not entirely unexpected, as HRP2, which binds HIV-1 IN with significantly lower affinity than LEDGF, is able to stimulate half-site integration *in vitro* to a similar extent [33]. Of note, because the ability of D366N LEDGF to bolster half-site strand transfer is severely repressed [34] (Figure 4B, lane 4), we argue that the stimulation of HIV-1 IN by LEDGF, and by EEE LEDGF, in particular, is strictly dependent on the direct protein-protein interaction. Concordantly, histidine and adenine auxotrophic AH109 yeast cells co-transformed with WT IN and EEE LEDGF Gal4 chimeras displayed a very slow growth phenotype on solid media lacking these nutrients, confirming a weak residual interaction (data not shown). In contrast, evidence for an interaction between D366N LEDGF and WT HIV-1 IN was not observed, even under these conditions [22].

In agreement with earlier observations [22], EIAV IN was highly competent for concerted integration in the presence of LEDGF (Figure 4C, lane 3). Notably, the concerted strand transfer activity of EIAV IN was severely reduced when EEE LEDGF was used (lane 4). At the same time, the trace levels of half-site activity were not significantly affected. These results indicated that the NTD–IBD interface bears a special significance for concerted lentiviral DNA integration. In the course of optimizing HIV-1 IN strand transfer conditions, we discovered that increasing donor DNA concentration greatly enhanced the yield of LEDGF-dependent concerted integration products (refer to Text S1 and Figure S4 for validation of the assay). This novel assay afforded a convenient means for studying the affects of mutations on LEDGF and HIV-1 IN function (Figure 4D). As expected, the D366N LEDGF mutant, severely defective for IN binding, failed to stimulate concerted integration (Figure 4D, compare lanes 4 and 5). Reaction products formed in the presence of AAA, ESE, and EEE LEDGF mutants show that successive addition of net negative charge at this location decreases the ability of the cofactor to stimulate concerted integration, with hardly any product visible with the EEE mutant (Figure 4D, lanes 6–8). However, in agreement with the data discussed above (Figure 4B), these mutants retained the ability to stimulate half-site integration. As expected, LEDGF mutants K360E and K392E displayed WT activity (Figure 4D, lanes 9 and 10).

Significantly, both KK and KKK IN mutants gained concerted integration activity in the presence of EEE LEDGF. Furthermore, both IN mutants, and most dramatically KKK IN, favored the mutant LEDGF form (Figure 4D, lanes 13–16). These results confirm that the mutant proteins are properly folded and that the effects observed are due to the modification of the protein-protein interface. They also suggest a possibility to engineer a gain of function HIV-1 IN mutant, active specifically in the presence of a complementary mutant of the host factor.

### The NTD–IBD Interface Is Important for LEDGF Cofactor Function During HIV-1 Infection

**Figure 5:**
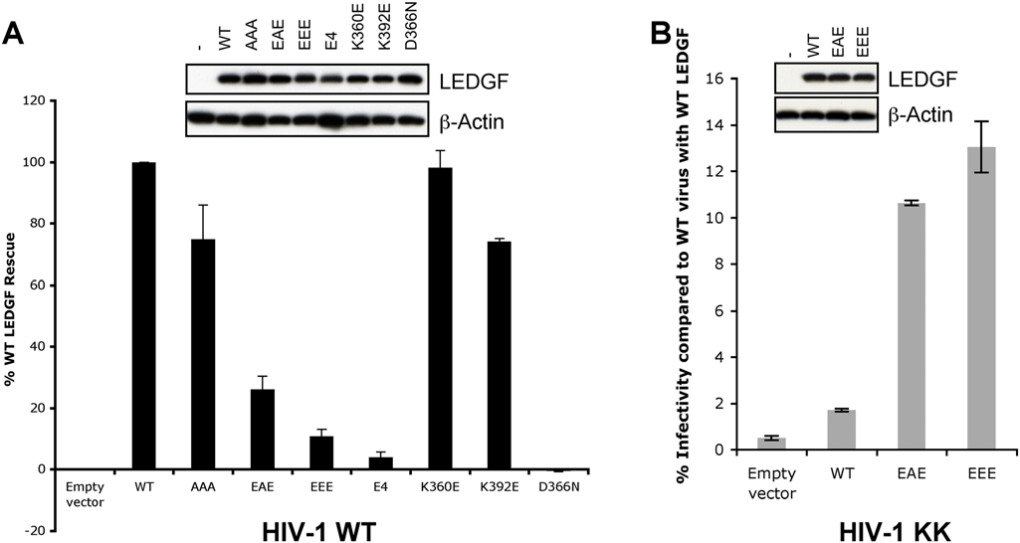
LEDGF Residues Lys-401, Arg-404, and Arg-405 Play a Crucial Role in HIV-1 Infection. (A) Relative infectivity of VSV G-pseudotyped WT HIV-Luc virus on *Ledgf*-null E2 MEFs transfected with human WT, AAA, EAE, EEE, E4, or D366N LEDGF expression vectors, as indicated. Infectivity of the virus in the presence of WT LEDGF was set to 100%; mean values and standard deviations combine data of two independent sets of transfections (each with infections performed in duplicate). (B) Infectivity of HIV-Luc bearing KK mutations in IN on E2 MEFs transfected with WT, EAE, or EEE LEDGF. Infectivity is expressed as a percentage of WT virus on the cells transfected with WT LEDGF. Western blots above the graphs show expression levels of LEDGF proteins and actin loading control.

To test the importance of the IBD-NTD interface in the context of viral replication, we used an established mouse LEDGF knockout model [29]. Although HIV-1 cannot complete its replication cycle in murine cells due to post-integration blocks, its reverse transcription and integration proceed normally and depend on LEDGF [29]. *Ledgf*-null mouse embryo fibroblasts (MEFs) transfected with a human LEDGF expression vector or its mutant forms were infected with single-round, vesicular stomatitis virus glycoprotein G (VSV-G)-pseudotyped HIV-1 vectors expressing a luciferase reporter gene (HIV-Luc), and the levels of luciferase activity in cell extracts were measured 44 h post infection. The WT and mutant LEDGF proteins were well-expressed, and endogenous mouse LEDGF protein, as predicted, was not detected in cells transfected with the empty vector (Figure 5A). WT LEDGF expression increased the level of knockout cell infection five to ten-fold as compared to cells carrying the empty vector. As expected [29], the D366N LEDGF mutant failed to stimulate the basal level of HIV-Luc infection. LEDGF AAA, K360E, and K392E by contrast supported similar levels of HIV-Luc infectivity as WT LEDGF, while its K401E/K402A/R405E (EAE, similar to ESE) and EEE mutants functioned at ∼25% and 10%, respectively (Figure 5A). An additional LEDGF mutant combining the EEE and K360E mutations, and therefore lacking the Lys-360:Glu-167 IBD–CCD salt bridge (E4, Figure 1B), supported the lowest level of infectivity (Figure 5A). These results tie in well with the *in vitro* interaction and activity data, extending the biological significance of the NTD-IBD interface.

Release and infectivity of HIV-1 mutants carrying substitutions at IN positions 6, 10, and 13 were impaired to various extents. The KKK variant was most affected, failing to support any appreciable infectivity under a variety of conditions. This result was not unexpected, as often subtler changes in IN grossly affect various HIV-1 replication steps [44]. The nature of the defects observed with mutant viruses will be elaborated elsewhere. As demonstrated above, the double mutant carrying substitutions at the more conserved NTD acidic positions (E10K/E13K) was able to functionally interact with EEE LEDGF. Although KK HIV-Luc supernatants harvested from transfected 293T cells contained approximately 30% of reverse transcriptase (RT) activity compared to WT, suggesting subtle release or maturation defects, in agreement with the *in vitro* data, this virus was infectious when presented with LEDGF EAE or EEE (Figure 5B). Significantly, KK HIV–Luc infectivity was negligible in the presence of WT LEDGF, generating luciferase levels comparable to the empty vector control (Figure 5B). In repeat experiments, the infectivity of the KK virus in the presence of LEDGF EEE varied between 3.5 and 40.8% (19.3±12.3% for *n* = 6 experiments) of WT HIV-Luc infectivity in the presence of WT LEDGF. These results confirm that modifications of the charge-charge NTD-IBD interface allow creation of viable gain of function IN mutants able to functionally interact with an engineered version of LEDGF.

## Discussion

In this work we extended the known lentiviral IN-LEDGF interface to include the interactions between the NTD of IN and the IBD of LEDGF. Since there is no evidence that the CTD of IN or LEDGF regions outside of the IBD are involved in the interaction, the contacts observed in our crystal structure may very well represent the entire IN-host factor interface. These novel structural details will invariably aid the development of inhibitors of the IN-LEDGF interaction. Intriguingly, both crystal forms of the HIV-2 IN_NTD+CCD_:LEDGF_IBD_ complex contained closed trimers of the IN_2_LEDGF substructure held together primarily by IN-IN contacts. Based on the three-fold symmetry of this assembly, we tentatively speculate that it could reflect packing arrangement of IN molecules within retroviral capsids, which too feature three-fold symmetry [45]. In both crystals the closed trimers further associated into a spherical particle containing twenty four IN chains, with their C-termini projecting inwards and the N-termini outwards. It remains to be determined if the higher order multimers of the IN_2_LEDGF substructure are biologically relevant. Such evidence could come, for example, by observing similar multimers in crystals of a divergent retroviral IN. Although we have not detected analogous large-sized complexes in solution, the calculated concentration of IN within retroviral capsids is very high [46], presenting an environment where it may very well adopt a paracrystalline state.

Mounting experimental evidence suggests that the active form of retroviral IN is a tetramer [13]–[15]. Based on a crystal structure of the HIV-1 IN_NTD+CCD_ fragment, Craigie and colleagues proposed a plausible model for the synaptic IN tetramer (dimer of dimers) [16]. Notably, the positions of the IN NTDs relative to the CCD dimer and the supporting NTD-CCD contacts observed in our HIV-2 IN_NTD+CCD_:LEDGF_IBD_ complex were seen in the earlier structure, where the NTDs mediated contacts between IN dimers [16]. One significant difference is that in the tetramer model, the NTD occupying the position primed for the interaction with the IBD is donated by the other IN dimer [16] (Figure S3A and S3B). Such domain swapping is quite common, representing one of the mechanisms for homomeric protein-protein interactions [47]. However, it is important to note an ambiguity of the NTD assignments in the HIV-1 IN_NTD+CCD_ structure, which lacked appreciable electron density for the NTD-CCD linkers [16]. Nevertheless, the NTD-CCD interface observed in the two independent structures is almost certainly biologically relevant. If IN dimers do indeed swap their NTDs during tetramerization, the LEDGF binding platform would include a CCD dimer and an NTD from a separate IN dimer (Figure S3C). Alternatively, LEDGF binding to an IN dimer would lock one NTD in the orientation primed for tetramerization (Figure S3D). In either case, upon binding, the co-factor would enhance the thermodynamic stability of the tetramer. It is a tetramer of IN that mediates synapsis of a pair of donor DNA molecules [15] and, concordantly, the NTD-IBD contacts uncovered here are specifically required for concerted DNA integration. The model can also explain the surprising ability of LEDGF to inhibit HIV-1 concerted integration under some *in vitro* conditions [42]. LEDGF binding to a dimer of IN would fixate either one or both NTDs, preventing them from functionally interacting with a second IN dimer (Figure S3). Thus, the concentration of IN, LEDGF and donor DNA substrate in the reaction mixture, as well as the order of component addition significantly influence the outcome of the reaction (Ref. [43] and data not shown). Of note, one recent study suggested that IN must exist in a lower multimeric state, likely a dimer, before interacting with the donor DNA for proper synaptic complex formation [48]. The enhanced concerted integration assay described herein will be very useful in future biochemical and structural studies of HIV-1 IN. Furthermore, other lentiviral INs, and in particular EIAV IN, carry out very efficient LEDGF-dependent concerted integration utilizing oligonucleotide donor DNA substrates under similar reaction conditions (data not shown).

Using complementary approaches we demonstrated that the NTD-IBD interface is important for the functional *Lentivirus*-host interaction. Compared to the lock-and-key CCD-IBD interface, the contacts involving the NTD are based on charge-charge interactions and therefore lend themselves to complementary reverse-charge engineering. Since LEDGF has been shown to target lentiviral integration to active transcription units [28]–[30], it has been speculated that modified versions of the host factor could be used to control integration site selection. Safety of retroviral vectors would be greatly improved if they could be directed towards specific pre-determined loci and away from proto-oncogenes [49]. In one recent work, a fusion of the DNA binding domain of bacteriophage λ repressor and the IBD of LEDGF targeted HIV-1 integration nearby λ operator sequences *in vitro* [50]. One fundamental problem can thwart practical application of such approaches. When delivered into the cell, the targeting factor, associated with a limited number of chromosomal loci, will have to compete with a vast excess of endogenous LEDGF for the incoming preintegration complex. Knockout or knockdown of endogenous LEDGF would unlikely be a practical or safe solution, especially considering its emerging role in epigenetics [18]. Here we demonstrated that an HIV-1 IN mutant carrying two reverse charge mutations within the NTD gained the ability to functionally interact with a modified version of LEDGF, while remaining basically unresponsive to the WT protein. Although the efficiency of the current system is somewhat modest, our results present a proof of principle that it is possible to engineer a viable complementary pair of IN and LEDGF mutants that could allow future development and practical applications of LEDGF-based lentiviral vector technologies.

## Materials and Methods

### Recombinant DNA

Compatible plasmids pCDF-HIV2-IN_NTD+CCD_ and pES-IBD-3C7 were used for co-expression of HIV-2 IN_NTD+CCD_ and LEDGF_IBD_ in bacteria. To obtain the former, a fragment encoding residues 1–209 of HIV-2 IN was inserted between *Nco*I and *Bam*HI sites of pCDF-Duet1 (Novagen); the latter was made by TA-cloning of a PCR fragment encoding LEDGF residues 347–471 with an internal human rhinovirus 14 (HRV14) 3 C protease cleavage site (LEVLFQGP, C-terminal to Val-435) into pET-SUMO (Invitrogen). pCPH6P-HIV1-IN, used for expression of full-length HIV-1 IN, was obtained by replacement of an *XmaI/XhoI* fragment encoding BIV IN in pCPH6P-BIV-IN [22] with a fragment coding for HIV-1_HXB2_ IN. Mutations were introduced into pCPH6P-HIV1-IN and pFT1-LEDGF [51] using the QuikChange procedure (Stratagene). For yeast two-hybrid assays, mutations were introduced into pCPY414-DBD-IN and pCPY426-AD-IBD [38] by swapping wild type IN and LEDGF fragments with their mutant forms. For virus infectivity assays, mutations were introduced into the *env*-deficient HIV-1 proviral clone pNLX.Luc(R-) encoding HIV-1 with a gene for firefly luciferase in place of Nef (HIV-Luc) and pIRES2-eGFP-LEDGF, as previously described [29],[52]. For production of SUMO protease, a PCR fragment encoding the catalytic core domain of *Saccharomyces cerevisiae* Ulp1 (residues 403–621) [53] was subcloned into pCPH6P-BIV-IN, replacing the BIV IN coding sequence to give pCPH6P-Ulp1CD. All DNA constructs made in this work were verified by sequencing to avoid inadvertent mutations.

### Protein Expression and Purification

To obtain HIV-2 IN_NTD+CCD_:LEDGF_IBD_ complex for crystallography, *Escherichia coli* PC2 cells [22] co-transformed with pCDF-HIV2-IN_NTD+CCD_ and pES-IBD-3C and grown in LB medium in the presence of 50 µg/ml kanamycin and 100 µg/ml spectinomycin to an A600 of ∼1.0 were supplemented with 50 µM ZnCl_2_ and induced with 0.3 mM isopropyl-β-D-thiogalactopyranoside. Following 4 h induction at 22°C, cells were harvested and stored at −80°C. For purification, cells were lysed by sonication in 1 M NaCl, 20 mM imidazole, 0.2 mM PMSF, 50 mM Tris-HCl, pH 7.4, and the protein complex was captured on Ni-NTA agarose (Qiagen). Following extensive washing the protein was eluted in 1 M NaCl, 200 mM imidazole, 50 mM Tris-HCl, pH 7.4. The His_6_-SUMO tag was cleaved by overnight incubation with SUMO protease at 7°C in the presence of 2 mM DTT. The sample was diluted with four volumes of 1 M NaCl, and the released His_6_-SUMO was depleted by absorption onto a 5-ml HisTrap column (GE Healthcare). Residues C-terminal to the IBD were removed by overnight digestion with HRV14 3 C protease in the presence of 10 mM DTT at 7°C. The complex was then purified by chromatography over a Superdex-200 column (GE Healthcare) in 1 M NaCl, 50 mM Tris-HCl, pH 7.4, concentrated to 17 mg/ml, supplemented with 10 mM DTT and 10% glycerol, and flash-frozen in liquid nitrogen.

Non-tagged wild type and mutant HIV-1 IN proteins used in strand transfer assays were produced in PC2 cells transformed with pCPH6P-HIV1-IN or its mutant forms as previously described [22]. The His_6_-tag was removed by digestion with HRV14 3 C protease. Non-tagged EIAV IN and C-terminally His_6_-tagged HIV-1, HIV-2, BIV, EIAV, and MVV IN proteins have been reported [22]. Wild type and mutant LEDGF proteins were made according to [51]. The SUMO protease Ulp1 catalytic domain fragment was produced in PC2 cells transformed with pCPH6P-Ulp1CD and purified as described in [53].

### Crystallization and Structure Determination

Vapor diffusion crystallization experiments were set up at 18°C in hanging drops by mixing 1 µl HIV-2 IN_NTD+CCD_:LEDGF_IBD_ complex at a concentration of 4 mg/ml in 20 mM Tris-HCl, pH 7.4, 300 mM NaCl, and 2 mM dithiothreitol (DTT) with 1 µl reservoir solution containing either 2.6 M sodium acetate, 0.1 M KI and 0.1 M Bis-Tris propane-HCl, pH 7.0 (form I) or 2.5 M sodium acetate, 10 mM MgCl_2_, and 0.1 M Bis-Tris propane-HCl, pH 7.0 (form II). Crystals of form I appeared within 24 h and reached the maximum size of ∼300×300×300 µm within 10–30 days, while those of form II appeared within a week and grew over several months to ∼150×150×150 µm. Both types of crystals were cryoprotected in 25% glycerol, 2.6 M sodium acetate, 10 mM MgCl_2_, 0.1 M Bis-Tris propane-HCl, pH 7.0. Diffraction data were collected to a resolution of 3.0 Å (form I) and 3.2 Å (form II) at the European Synchrotron Radiation Facility (ESRF) beamline ID23-1 at 100 K. The data were processed using *MOSFLM* [54] and *SCALA* [55] part of the *CCP4* project [56].

Crystal form I belonged to the space group P321 with unit cell parameters a = b = 210.5 Å, c = 162.6 Å, α = β = 90°, and γ = 120°. The structure was solved by molecular replacement with *MOLREP* [57] using three individual search models in the following order. First, three dimers of CCDs (chains A and B from 2b4j) were located, forming a trimer of dimers, followed by a single IBD molecule (chain C from 2b4j) per CCD dimer, and finally the NTDs (chain A residues 1–45 from 1k6y) [16],[36]. After rigid body refinement, it became clear that a fourth IN dimer with corresponding IBD molecule was located out of the plane of the original trimer of dimers, and that this new dimer formed a similar trimer of dimers via the crystallographic three-fold axis. The ASU contained twelve protein chains and over 70% solvent. The structure was refined using *REFMAC* [58] and *PHENIX* [59], including translation, libration and screw (TLS) refinement, with manual model building in *COOT* [60].

Form II crystals belonged to the space group P2_1_2_1_2_1_ with unit cell parameters a = 201.4 Å, b = 202.5 Å, c = 280.5 Å, and α = β = γ = 90°. A high degree of NCS was expected due to the large unit cell. Therefore, to reduce potential bias in R_free_ estimation, the test reflection set was chosen in thin shells using *SFTOOLS*, part of the *CCP4* program suite [56]. The structure was solved by molecular replacement in *PHASER* [61], using a search model containing the dimeric IN assembly plus an associated LEDGF chain as observed in form I. The structure was refined using simulated annealing in *PHENIX* and restrained refinement in *REFMAC*, imposing tight 12-fold NCS restraints. Positive Fo-Fc density was observed at the known binding sites for zinc and magnesium and the corresponding atom was added to the structure. Details on data collection and refinement statistics are shown in Table 1. Diffraction data and the resulting structure derived from crystal form II were deposited to the protein databank (PDB ID 3f9k), and those for form I are available upon request.

### Protein–Protein Interaction and Strand Transfer Activity Assays

His_6_-tag pull-down and yeast two-hybrid assays were performed as described previously [22],[34],[38]. The indicator *S. cerevisiae* strains Y187 and AH109 were from BD Biosciences. Untagged, recombinant INs were used in all strand transfer assays. HIV-1 and EIAV integration assays with the respective 500-bp and 225-bp RU5 donor DNA substrates were carried out as previously described [22]. HIV-1 donor DNA was obtained as a PCR product using Pfu DNA polymerase (Stratagene) with the primer pair 5′-GGACTGAGGGGCCTGAAATGAGC/5′-ACTGTTGGGTGTTCTTCACCGCCCC GCGAGCT and pU3U5 template; primers 5′-TTAAGTTGGGTAACGCCAGG/5′-ACT GTAGGATCTCGAACAGAC and pU3U5-EIAV template were used to make the EIAV donor [22],[46].

For enhanced HIV-1 concerted integration assays, donor DNA was prepared by annealing DNA oligonucleotides 5′-CCTTTTAGTCAGTGTGGAAAATCTCTAGCA or 5′-CCTTTTAGTCAGTGTGGAAAATCTCTAGCAGT and 5′-ACTGCTAGAGATTTT CCACACTGACTAAAAGG to create a 32 bp mimic of the pre-processed or non-processed HIV-1 U5 cDNA terminus, respectively. Two µl HIV-1 IN in 750 mM NaCl, 2 mM DTT, 20 mM Tris-HCl, pH 7.4 (DB) was added to 36 µl master mix containing 0.55 µM donor DNA and 0.30 µg supercoiled pGEM-9Zf(-) target DNA in 25.3 mM NaCl, 5.5 mM MgSO_4_, 11 mM DTT, 4.4 µM ZnCl_2_, 22 mM HEPES-NaOH, pH 7.4. Following a 3–5 min pre-incubation at room temperature, reactions were supplemented with 2 µl LEDGF in DB and allowed to proceed at 37°C for 30 min. The final concentrations of HIV-1 IN and LEDGF were both 0.6 µM. The reactions were stopped by addition of 25 mM EDTA and 0.5% SDS. The products deproteinized by digestion with 30 µg Proteinase K for 1 h at 37°C and ethanol precipitation were resolved by electrophoresis in 1.5% agarose gels and detected using ethidium bromide.

For sequencing analysis, reaction products migrating as a band of ∼3 kb were isolated from a 1.5% agarose gel and converted into fully double stranded forms by treatment with Φ29 DNA polymerase (New England Biolabs) in the presence of 500 µM dNTPs. The DNA was then 5′-phosphorylated and ligated to a blunt-ended 1.2-kb PCR fragment spanning the Tn5 aminoglycoside-3′-*O*-phosphotransferase gene flanked by *Kpn*I sites [22]. Competent DH5α *E. coli* cells were transformed with the ligation mixture. Plasmids were isolated from individual kanamycin-resistant colonies, and those releasing fragments of expected sizes (∼3 and 1.2 kb) upon digestion with *Kpn*I, were sequenced using primers annealing within the Tn5-derived fragment.

### Infectivity Assays

Single cycle infectivity assays were done as described elsewhere [29],[62]. Briefly, VSV-G-pseudotyped HIV-Luc carrying various IN alleles generated by transfecting 293T cells were titered using a ^32^P-based reverse transcriptase (RT) assay. The *Ledgf*-null E2(−/−) mouse embryo fibroblasts (MEFs) transformed with simian virus 40 large T antigen were previously described [29]. Cells transfected with empty, WT, or mutant LEDGF pIRES2-eGFP expression vectors and sorted by FACS to enrich the GFP-positive population were lysed for western blot analyses or plated for infections. 10 h after plating, the cells were infected with equal RT-cpm of HIV-Luc variants. Cells were lysed and luciferase activity relative to the total protein content of the lysates was measured 44 h post infection.

## Supporting Information

Text S1 Validation of LEDGF-Dependent Concerted HIV-1 IN Strand Transfer Activity
(0.04 MB DOC)

Figure S1 Examples of Electron Density for Different Regions of the Structure. (A) The CCD-IBD interface with crucial LEDGF residues Asp-366 and Ile-365 labeled. In all panels the 2fo-fc electron density contoured at 1.5 σ level is shown as chicken wire in blue, and protein residues are represented as sticks. Coloring of carbon atoms is related to their chain (green - IN chain A, cyan - IN chain B, and pink - LEDGF chain C); nitrogen, oxygen, and sulfur are blue, red, and yellow, respectively. (B) The HHCC motif, including a coordinated Zn atom (gray sphere). (C) The native side-chain and corresponding electron density of HIV-2 IN residue Phe-185.
(6.93 MB TIF)

Figure S2 Stereo Image Showing Details of the HIV-2 IN CCD-LEDGF IBD Interface. Interacting residues are shown as sticks and are labeled. Cartoon and carbon atoms are colored according to their chain (green - IN chain A, cyan - IN chain B, and pink - LEDGF chain C). Hydrogen bonds between Asp-366 and the main chain amides of IN residues Asn-170 and Thr-171 are shown as black dashes.
(1.28 MB TIF)

Figure S3 A Model for IN Tetramerization via Inter-Dimer NTD Swapping. (A) The IN_2_LEDGF substructure is shown in green, cyan, and pink (as in Figure 1A). The NTD colored dark blue belongs to a separate IN_2_LEDGF unit, its contacts with the IBD stabilize the closed trimer (Figure 1B). (B) The structure of uncomplexed HIV-1 IN_NTD+CCD_ (PDB ID 1k6y), crystallized as a tetramer (dimer of dimers). One dimer of IN (green and cyan) is shown with the CCDs in the same orientation as in panel A, with the tetramer completed by the dimer colored yellow and orange. The yellow NTD enclosed in an oval is in the same position and orientation as the green NTD in (A), while the orange NTD (circled) is in a similar position but a different orientation to the dark blue NTD in (A). (C) Schematic diagram of the proposed assembly and domain interactions within the active synaptic complex (IN CTDs are not shown). Coloring of the IN and IBD molecules is same as in (A) and (B); large circles represent the IN CCDs, small circles the NTDs, and parallelograms the IBDs. NTDs are connected to the same-chain CCDs by flexible hinges (black curves). Association of the two dimers during synaptic complex assembly involves a swap of an NTD from each dimer to interact with a CCD from the opposing dimer. When loaded, LEDGF would engage the CCDs from one dimer and an NTD from another, effectively stabilizing the complex. Further, it is also speculatively possible that the other NTD interacts with the IBD, as seen with the dark blue NTD in (A). (D) Alternative model for the assembly of the synaptic complex in which there is no NTD swap. In this case the IBD of LEDGF stabilizes the synaptic complex by locking the NTDs in the correct orientation for tetramerization.
(0.81 MB JPG)

Figure S4 Validation of LEDGF-Dependent Concerted HIV-1 IN Strand Transfer Activity. (A) Increasing input of donor DNA favors concerted integration. Lane 1 contains a mock sample, where IN and LEDGF were omitted; lane 2 shows activity in the absence of LEDGF; lane 3 contained both LEDGF and IN, but no donor DNA. Lanes 4–9: HIV-1 IN was incubated with 1 µM-4 nM (as indicated) blunt 32-bp donor DNA mimicking the U5 HIV-1 cDNA end and supercoiled target DNA in the presence of LEDGF. (B) HIV-1 IN strand transfer inhibitor MK0518 (Raltegravir) effectively blocks accumulation of full- and half-site reaction products. Lanes 4–9: HIV-1 IN was incubated with 0.5 µM pre-processed 32-bp donor DNA in the presence of LEDGF and MK0518 at final concentrations of 1,400 µM (lane 4), 140 µM (lane 5), 14 µM (lane 6), 1.4 µM (lane 7), 0.14 µM (lane 8), or 0.014 µM (lane 9). Almost complete suppression of half- and full-site integration is achieved at 1.4 µM MK0518, which is comparable to the concentration of IN (0.8 µM) used in this experiment. The inhibitor was not present in lane 3. Lane 1 contained a mock sample without IN and LEDGF; the cofactor was omitted from the reaction in lane 2. Migration positions of DNA size standards, DNA substrate, open circular (o.c.), and supercoiled (s.c.) target DNA forms, half- and concerted (full-site) products are indicated. (C) 2-D agarose gel analysis of reaction products formed under conditions of enhanced concerted integration. DNA species obtained from incubation of pre-processed 32-bp donor DNA and supercoiled pGEM target in the presence of HIV-1 IN and LEDGF (lane 1), IN alone (lane 2), or in the absence of both proteins (lane 3) were separated in 0.6% agarose along with 1-kb ladder DNA (lane L) (top gel). A lane with a sample identical to that in lane 1 was excised from the gel. In this gel slice, two wells were created for parallel separation of the 1-kb DNA ladder and another aliquot of sample 1. The DNA was then separated in the perpendicular direction in a 1.6% agarose gel and visualized with ethidium bromide. Projected migrations of the linear DNA standards are indicated with red crosses. Note migration of the full-site (FS) product along the arc defined by the linear DNA size standards, while circular DNA species (half-site [HS], o.c. and s.c. target DNA) appear above the arc. Products of multiple full-site events are expected to result in a gamut of linear DNA species of variable lengths migrating as smears in agarose gels; akin to the full-site product, these species distribute along the arc.
(2.33 MB TIF)

## References

[1] Lewinski MK, Bushman FD (2005). Retroviral DNA integration–mechanism and consequences.. Adv Genet.

[2] Craigie R, Craig NL, Craigie R, Gellert M, Lambowitz AM (2002). Retroviral DNA Integration.. Mobile DNA II.

[3] Bushman FD, Engelman A, Palmer I, Wingfield P, Craigie R (1993). Domains of the integrase protein of human immunodeficiency virus type 1 responsible for polynucleotidyl transfer and zinc binding.. Proc Natl Acad Sci USA.

[4] Engelman A, Bushman FD, Craigie R (1993). Identification of discrete functional domains of HIV-1 integrase and their organization within an active multimeric complex.. EMBO J.

[5] Engelman A, Craigie R (1992). Identification of conserved amino acid residues critical for human immunodeficiency virus type 1 integrase function in vitro.. J Virol.

[6] Dyda F, Hickman AB, Jenkins TM, Engelman A, Craigie R (1994). Crystal structure of the catalytic domain of HIV-1 integrase: similarity to other polynucleotidyl transferases.. Science.

[7] Goldgur Y, Dyda F, Hickman AB, Jenkins TM, Craigie R (1998). Three new structures of the core domain of HIV-1 integrase: an active site that binds magnesium.. Proc Natl Acad Sci USA.

[8] Yang W, Lee JY, Nowotny M (2006). Making and breaking nucleic acids: two-Mg2+−ion catalysis and substrate specificity.. Mol Cell.

[9] Cai M, Zheng R, Caffrey M, Craigie R, Clore GM (1997). Solution structure of the N-terminal zinc binding domain of HIV-1 integrase.. Nat Struct Biol.

[10] Eijkelenboom AP, van den Ent FM, Vos A, Doreleijers JF, Hard K (1997). The solution structure of the amino-terminal HHCC domain of HIV-2 integrase: a three-helix bundle stabilized by zinc.. Curr Biol.

[11] Eijkelenboom AP, Lutzke RA, Boelens R, Plasterk RH, Kaptein R (1995). The DNA-binding domain of HIV-1 integrase has an SH3-like fold.. Nat Struct Biol.

[12] Lodi PJ, Ernst JA, Kuszewski J, Hickman AB, Engelman A (1995). Solution structure of the DNA binding domain of HIV-1 integrase.. Biochemistry (Mosc).

[13] Bao KK, Wang H, Miller JK, Erie DA, Skalka AM (2003). Functional oligomeric state of avian sarcoma virus integrase.. J Biol Chem.

[14] Faure A, Calmels C, Desjobert C, Castroviejo M, Caumont-Sarcos A (2005). HIV-1 integrase crosslinked oligomers are active in vitro.. Nucleic Acids Res.

[15] Li M, Mizuuchi M, Burke TR, Craigie R (2006). Retroviral DNA integration: reaction pathway and critical intermediates.. EMBO J.

[16] Wang JY, Ling H, Yang W, Craigie R (2001). Structure of a two-domain fragment of HIV-1 integrase: implications for domain organization in the intact protein.. EMBO J.

[17] Engelman A, Cherepanov P (2008). The lentiviral integrase binding protein LEDGF/p75 and HIV-1 replication.. PLoS Pathog.

[18] Yokoyama A, Cleary ML (2008). Menin critically links MLL proteins with LEDGF on cancer-associated target genes.. Cancer Cell.

[19] Singh DP, Fatma N, Kimura A, Chylack LT, Shinohara T (2001). LEDGF binds to heat shock and stress-related element to activate the expression of stress-related genes.. Biochem Biophys Res Commun.

[20] Wu X, Daniels T, Molinaro C, Lilly MB, Casiano CA (2002). Caspase cleavage of the nuclear autoantigen LEDGF/p75 abrogates its pro-survival function: implications for autoimmunity in atopic disorders.. Cell Death Differ.

[21] Cherepanov P, Maertens G, Proost P, Devreese B, Van Beeumen J (2003). HIV-1 integrase forms stable tetramers and associates with LEDGF/p75 protein in human cells.. J Biol Chem.

[22] Cherepanov P (2007). LEDGF/p75 interacts with divergent lentiviral integrases and modulates their enzymatic activity in vitro.. Nucleic Acids Res.

[23] Busschots K, Vercammen J, Emiliani S, Benarous R, Engelborghs Y (2005). The interaction of LEDGF/p75 with integrase is lentivirus-specific and promotes DNA binding.. J Biol Chem.

[24] Llano M, Vanegas M, Fregoso O, Saenz D, Chung S (2004). LEDGF/p75 determines cellular trafficking of diverse lentiviral but not murine oncoretroviral integrase proteins and is a component of functional lentiviral preintegration complexes.. J Virol.

[25] Emiliani S, Mousnier A, Busschots K, Maroun M, Van Maele B (2005). Integrase mutants defective for interaction with LEDGF/p75 are impaired in chromosome tethering and HIV-1 replication.. J Biol Chem.

[26] Llano M, Vanegas M, Hutchins N, Thompson D, Delgado S (2006). Identification and characterization of the chromatin-binding domains of the HIV-1 integrase interactor LEDGF/p75.. J Mol Biol.

[27] Maertens G, Cherepanov P, Pluymers W, Busschots K, De Clercq E (2003). LEDGF/p75 is essential for nuclear and chromosomal targeting of HIV-1 integrase in human cells.. J Biol Chem.

[28] Ciuffi A, Llano M, Poeschla E, Hoffmann C, Leipzig J (2005). A role for LEDGF/p75 in targeting HIV DNA integration.. Nat Med.

[29] Shun MC, Raghavendra NK, Vandegraaff N, Daigle JE, Hughes S (2007). LEDGF/p75 functions downstream from preintegration complex formation to effect gene-specific HIV-1 integration.. Genes Dev.

[30] Marshall HM, Ronen K, Berry C, Llano M, Sutherland H (2007). Role of PSIP1/LEDGF/p75 in lentiviral infectivity and integration targeting.. PLoS ONE.

[31] Llano M, Saenz DT, Meehan A, Wongthida P, Peretz M (2006). An essential role for LEDGF/p75 in HIV integration.. Science.

[32] Hombrouck A, De Rijck J, Hendrix J, Vandekerckhove L, Voet A (2007). Virus evolution reveals an exclusive role for LEDGF/p75 in chromosomal tethering of HIV.. PLoS Pathog.

[33] Cherepanov P, Devroe E, Silver PA, Engelman A (2004). Identification of an evolutionarily conserved domain in human lens epithelium-derived growth factor/transcriptional co-activator p75 (LEDGF/p75) that binds HIV-1 integrase.. J Biol Chem.

[34] Cherepanov P, Sun ZY, Rahman S, Maertens G, Wagner G (2005). Solution structure of the HIV-1 integrase-binding domain in LEDGF/p75.. Nat Struct Mol Biol.

[35] Vanegas M, Llano M, Delgado S, Thompson D, Peretz M (2005). Identification of the LEDGF/p75 HIV-1 integrase-interaction domain and NLS reveals NLS-independent chromatin tethering.. J Cell Sci.

[36] Cherepanov P, Ambrosio AL, Rahman S, Ellenberger T, Engelman A (2005). Structural basis for the recognition between HIV-1 integrase and transcriptional coactivator p75.. Proc Natl Acad Sci USA.

[37] Burke CJ, Sanyal G, Bruner MW, Ryan JA, LaFemina RL (1992). Structural implications of spectroscopic characterization of a putative zinc finger peptide from HIV-1 integrase.. J Biol Chem.

[38] Rahman S, Lu R, Vandegraaff N, Cherepanov P, Engelman A (2007). Structure-based mutagenesis of the integrase-LEDGF/p75 interface uncouples a strict correlation between in vitro protein binding and HIV-1 fitness.. Virology.

[39] Sinha S, Grandgenett DP (2005). Recombinant human immunodeficiency virus type 1 integrase exhibits a capacity for full-site integration in vitro that is comparable to that of purified preintegration complexes from virus-infected cells.. J Virol.

[40] Li M, Craigie R (2005). Processing of viral DNA ends channels the HIV-1 integration reaction to concerted integration.. J Biol Chem.

[41] Valkov E, Gupta SS, Hare S, Helander A, Roversi P (2008). Functional and structural characterization of the integrase from the prototype foamy virus.. Nucleic Acids Res.

[42] Raghavendra NK, Engelman A (2007). LEDGF/p75 interferes with the formation of synaptic nucleoprotein complexes that catalyze full-site HIV-1 DNA integration in vitro: implications for the mechanism of viral cDNA integration.. Virology.

[43] Pandey KK, Sinha S, Grandgenett DP (2007). Transcriptional coactivator LEDGF/p75 modulates human immunodeficiency virus type 1 integrase-mediated concerted integration.. J Virol.

[44] Engelman A (1999). In vivo analysis of retroviral integrase structure and function.. Adv Virus Res.

[45] Ganser-Pornillos BK, Yeager M, Sundquist WI (2008). The structural biology of HIV assembly.. Curr Opin Struct Biol.

[46] Cherepanov P, Surratt D, Toelen J, Pluymers W, Griffith J (1999). Activity of recombinant HIV-1 integrase on mini-HIV DNA.. Nucleic Acids Res.

[47] Rousseau F, Schymkowitz JW, Itzhaki LS (2003). The unfolding story of three-dimensional domain swapping.. Structure.

[48] Lesbats P, Metifiot M, Calmels C, Baranova S, Nevinsky G (2008). In vitro initial attachment of HIV-1 integrase to viral ends: control of the DNA specific interaction by the oligomerization state.. Nucleic Acids Res.

[49] Hacein-Bey-Abina S, Von Kalle C, Schmidt M, McCormack MP, Wulffraat N (2003). LMO2-associated clonal T cell proliferation in two patients after gene therapy for SCID-X1.. Science.

[50] Ciuffi A, Diamond TL, Hwang Y, Marshall HM, Bushman FD (2006). Modulating target site selection during human immunodeficiency virus DNA integration in vitro with an engineered tethering factor.. Hum Gene Ther.

[51] Turlure F, Maertens G, Rahman S, Cherepanov P, Engelman A (2006). A tripartite DNA-binding element, comprised of the nuclear localization signal and two AT-hook motifs, mediates the association of LEDGF/p75 with chromatin in vivo.. Nucleic Acids Res.

[52] Lu R, Limon A, Devroe E, Silver PA, Cherepanov P (2004). Class II integrase mutants with changes in putative nuclear localization signals are primarily blocked at a postnuclear entry step of human immunodeficiency virus type 1 replication.. J Virol.

[53] Mossessova E, Lima CD (2000). Ulp1-SUMO crystal structure and genetic analysis reveal conserved interactions and a regulatory element essential for cell growth in yeast.. Mol Cell.

[54] Leslie AGW (1992). Recent changes to the MOSFLM package for processing film and image plate data.. Joint CCP4+ESF-EAMCB Newsletter on Protein Crystallography.

[55] Evans PR (1993). Data reduction.. Proceedings of CCP4 Study Weekend, 1993, on Data Collection & Processing.

[56] CCP4 (1994). The CCP 4 suite: programs for protein crystallography.. Acta Crystallography D.

[57] Vagin A (1997). MOLREP: an automated program for molecular replacement.. J Appl Cryst.

[58] Murshudov GN, Vagin AA, Dodson EJ (1997). Refinement of macromolecular structures by the maximum-likelihood method.. Acta Crystallogr D Biol Crystallogr.

[59] Adams PD, Grosse-Kunstleve RW, Hung LW, Ioerger TR, McCoy AJ (2002). PHENIX: building new software for automated crystallographic structure determination.. Acta Crystallogr D Biol Crystallogr.

[60] Emsley P, Cowtan K (2004). Coot: model-building tools for molecular graphics.. Acta Crystallogr D Biol Crystallogr.

[61] McCoy AJ, Grosse-Kunstleve RW, Adams PD, Winn MD, Storoni LC (2007). Phaser crystallographic software.. J Appl Cryst.

[62] Shun MC, Daigle JE, Vandegraaff N, Engelman A (2007). Wild-type levels of human immunodeficiency virus type 1 infectivity in the absence of cellular emerin protein.. J Virol.

