# Supplementary material for: A Novel Co-Crystal Structure Affords the Design of Gain-of-Function Lentiviral Integrase Mutants in the Presence of Modified PSIP1/LEDGF/p75
Source: PLoS Pathog. 2009 Jan 9;5(1):e1000259. doi: 10.1371/journal.ppat.1000259 (PMC2606027; doi:10.1371/journal.ppat.1000259)
Supplement: Text S1 — Validation of LEDGF-Dependent Concerted HIV-1 IN Strand Transfer Activity (0.04 MB DOC) [file ppat.1000259.s001.doc]

**Supporting Text S1.**

**Validation of LEDGF-Dependent Concerted HIV-1 IN Strand Transfer Activity**

In the presence of LEDGF, HIV-1 IN is quite prone for concerted integration when presented with 40-1,000 nM blunt or pre-processed donor 32-bp HIV-1 U5 end mimic (Figure S4). Accumulation of DNA species migrating close to the predicted position for products of full-site integration (2,976 bp) was dependent on the presence of donor DNA, IN, and LEDGF (Figure S4A) and was responsive to HIV‑1 IN strand transfer inhibitor MK0158 [1] (Figure S4B). The migration of these products in agarose gels of different strengths [2,3] was close to that expected for linear DNA species (Figure S4C). To further verify that these species are *de facto* products of concerted integration, DNA material migrating in 1.5% agarose gels at ~3,000 bp was isolated and cloned. Sequencing of the resulting clones revealed that 83% (*n*=18) contained pairs of donor DNA sequences inserted with 5 bp duplications of the target DNA sequence, confirming the nature of the products. It is important to note that although increased donor concentration helps to visualize concerted integration products, it does not suppress the half site process. Due to the small size of the donor, the half site products co-migrate with open circular target DNA. Under these conditions, the supercoiled target DNA becomes a limiting factor (Figure S4A, note its depletion in lanes 4-6). This leads to the re-targeting of the full-site integration products, resulting in smearing of the final full-site product band.

**Supporting References**

1. Summa V, Petrocchi A, Bonelli F, Crescenzi B, Donghi M, et al. (2008) Discovery of raltegravir, a potent, selective orally bioavailable HIV-integrase inhibitor for the treatment of HIV-AIDS infection. J Med Chem 51: 5843-5855.

2. Friedman KL, Brewer BJ (1995) Analysis of replication intermediates by two-dimensional agarose gel electrophoresis. Methods Enzymol 262: 613-627.

3. Raghavendra NK, Engelman A (2007) LEDGF/p75 interferes with the formation of synaptic nucleoprotein complexes that catalyze full-site HIV-1 DNA integration *in vitro*: implications for the mechanism of viral cDNA integration. Virology 360: 1-5.
