# Supplementary figures and images for: A Novel Co-Crystal Structure Affords the Design of Gain-of-Function Lentiviral Integrase Mutants in the Presence of Modified PSIP1/LEDGF/p75
Source: PLoS Pathog. 2009 Jan 9;5(1):e1000259. doi: 10.1371/journal.ppat.1000259 (PMC2606027; doi:10.1371/journal.ppat.1000259)

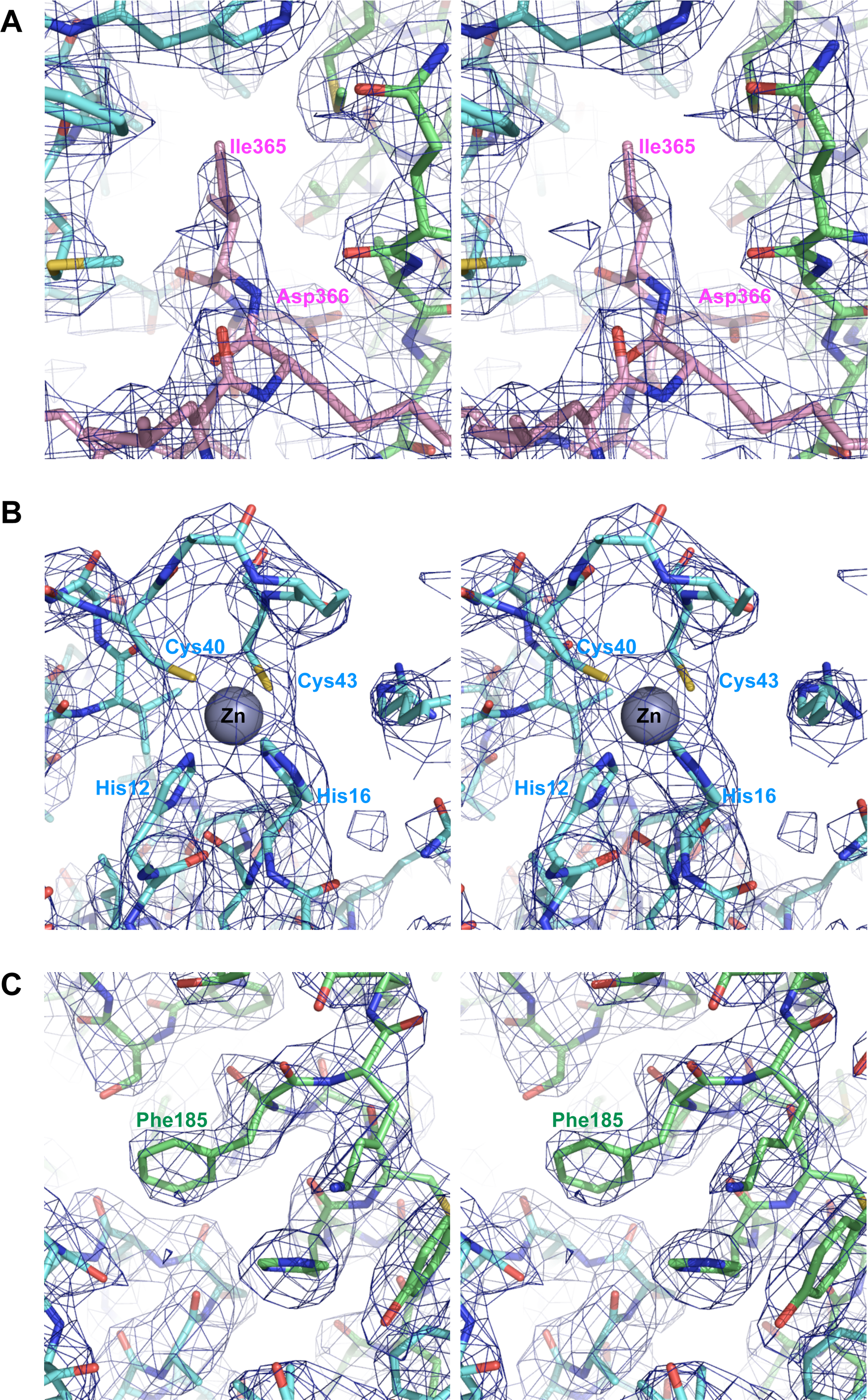

Supplement: Figure S1 — Examples of Electron Density for Different Regions of the Structure. (A) The CCD-IBD interface with crucial LEDGF residues Asp-366 and Ile-365 labeled. In all panels the 2fo-fc electron density contoured at 1.5 σ level is shown as chicken wire in blue, and protein residues are represented as sticks. Coloring of carbon atoms is related to their chain (green - IN chain A, cyan - IN chain B, and pink - LEDGF chain C); nitrogen, oxygen, and sulfur are blue, red, and yellow, respectively. (B) The HHCC motif, including a coordinated Zn atom (gray sphere). (C) The native side-chain and corresponding electron density of HIV-2 IN residue Phe-185. (6.93 MB TIF) [file ppat.1000259.s002.tif]

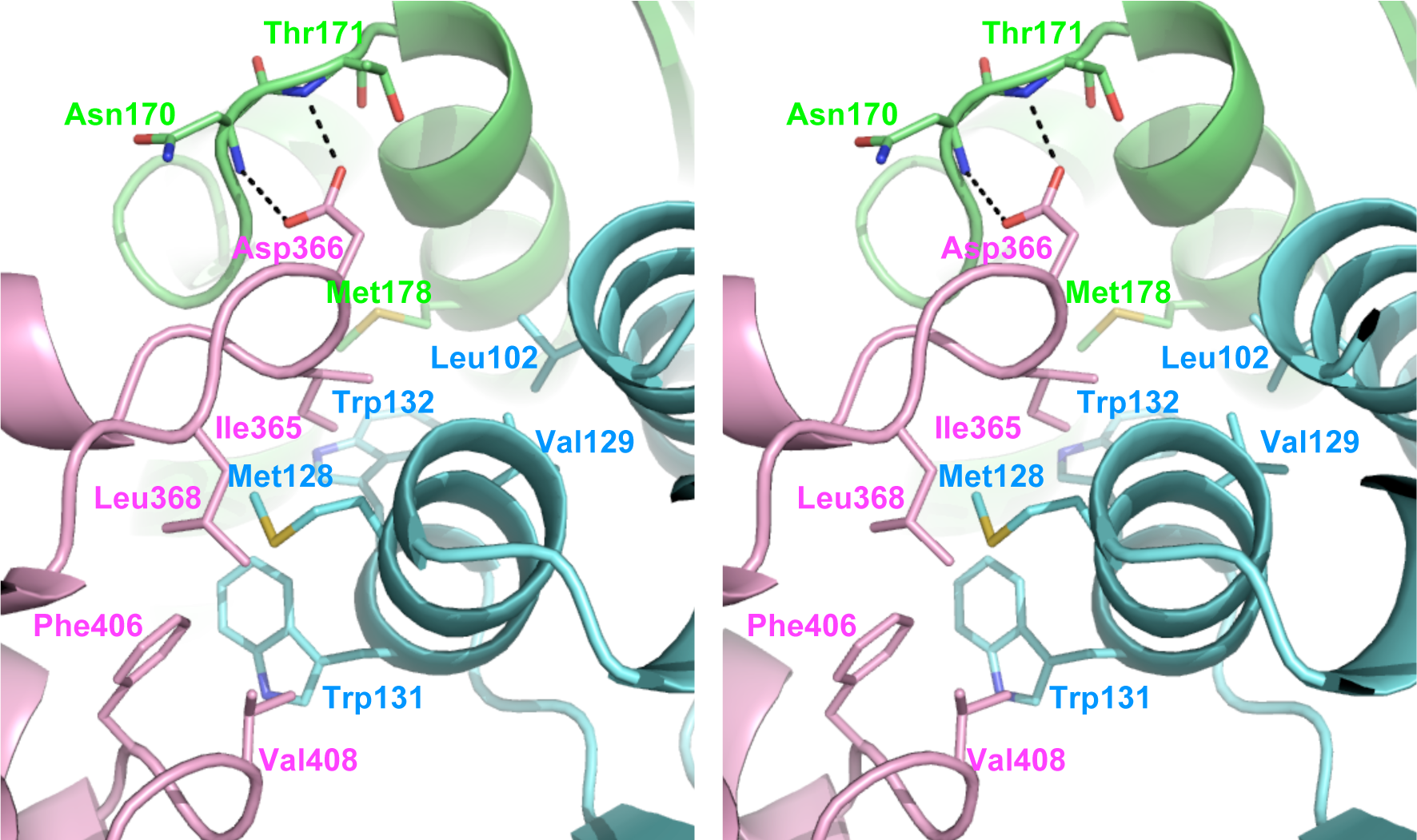

Supplement: Figure S2 — Stereo Image Showing Details of the HIV-2 IN CCD-LEDGF IBD Interface. Interacting residues are shown as sticks and are labeled. Cartoon and carbon atoms are colored according to their chain (green - IN chain A, cyan - IN chain B, and pink - LEDGF chain C). Hydrogen bonds between Asp-366 and the main chain amides of IN residues Asn-170 and Thr-171 are shown as black dashes. (1.28 MB TIF) [file ppat.1000259.s003.tif]

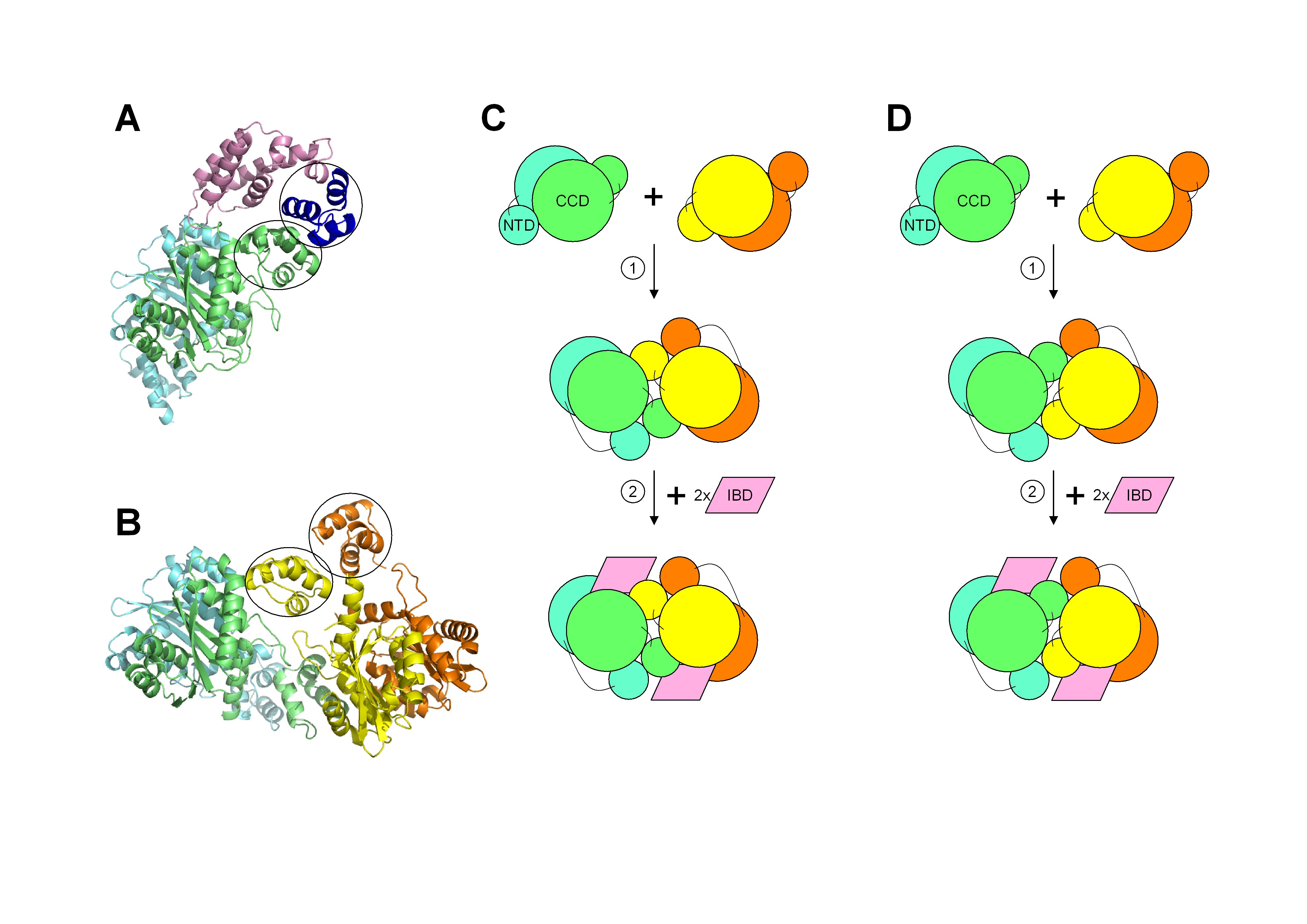

Supplement: Figure S3 — A Model for IN Tetramerization via Inter-Dimer NTD Swapping. (A) The IN2LEDGF substructure is shown in green, cyan, and pink (as in Figure 1A). The NTD colored dark blue belongs to a separate IN2LEDGF unit, its contacts with the IBD stabilize the closed trimer (Figure 1B). (B) The structure of uncomplexed HIV-1 INNTD+CCD (PDB ID 1k6y), crystallized as a tetramer (dimer of dimers). One dimer of IN (green and cyan) is shown with the CCDs in the same orientation as in panel A, with the tetramer completed by the dimer colored yellow and orange. The yellow NTD enclosed in an oval is in the same position and orientation as the green NTD in (A), while the orange NTD (circled) is in a similar position but a different orientation to the dark blue NTD in (A). (C) Schematic diagram of the proposed assembly and domain interactions within the active synaptic complex (IN CTDs are not shown). Coloring of the IN and IBD molecules is same as in (A) and (B); large circles represent the IN CCDs, small circles the NTDs, and parallelograms the IBDs. NTDs are connected to the same-chain CCDs by flexible hinges (black curves). Association of the two dimers during synaptic complex assembly involves a swap of an NTD from each dimer to interact with a CCD from the opposing dimer. When loaded, LEDGF would engage the CCDs from one dimer and an NTD from another, effectively stabilizing the complex. Further, it is also speculatively possible that the other NTD interacts with the IBD, as seen with the dark blue NTD in (A). (D) Alternative model for the assembly of the synaptic complex in which there is no NTD swap. In this case the IBD of LEDGF stabilizes the synaptic complex by locking the NTDs in the correct orientation for tetramerization. (0.81 MB JPG) [file ppat.1000259.s004.jpg]

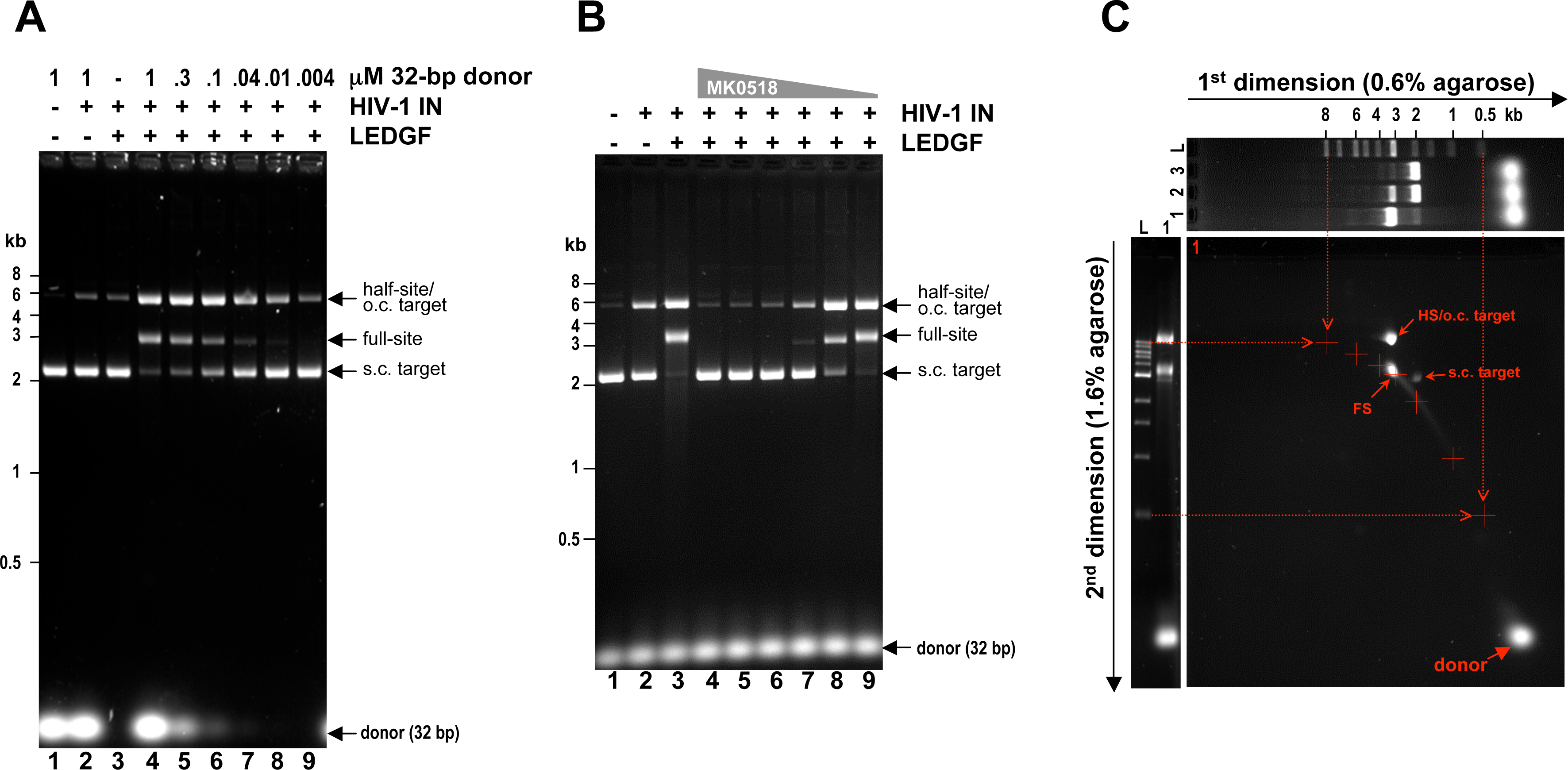

Supplement: Figure S4 — Validation of LEDGF-Dependent Concerted HIV-1 IN Strand Transfer Activity. (A) Increasing input of donor DNA favors concerted integration. Lane 1 contains a mock sample, where IN and LEDGF were omitted; lane 2 shows activity in the absence of LEDGF; lane 3 contained both LEDGF and IN, but no donor DNA. Lanes 4–9: HIV-1 IN was incubated with 1 µM-4 nM (as indicated) blunt 32-bp donor DNA mimicking the U5 HIV-1 cDNA end and supercoiled target DNA in the presence of LEDGF. (B) HIV-1 IN strand transfer inhibitor MK0518 (Raltegravir) effectively blocks accumulation of full- and half-site reaction products. Lanes 4–9: HIV-1 IN was incubated with 0.5 µM pre-processed 32-bp donor DNA in the presence of LEDGF and MK0518 at final concentrations of 1,400 µM (lane 4), 140 µM (lane 5), 14 µM (lane 6), 1.4 µM (lane 7), 0.14 µM (lane 8), or 0.014 µM (lane 9). Almost complete suppression of half- and full-site integration is achieved at 1.4 µM MK0518, which is comparable to the concentration of IN (0.8 µM) used in this experiment. The inhibitor was not present in lane 3. Lane 1 contained a mock sample without IN and LEDGF; the cofactor was omitted from the reaction in lane 2. Migration positions of DNA size standards, DNA substrate, open circular (o.c.), and supercoiled (s.c.) target DNA forms, half- and concerted (full-site) products are indicated. (C) 2-D agarose gel analysis of reaction products formed under conditions of enhanced concerted integration. DNA species obtained from incubation of pre-processed 32-bp donor DNA and supercoiled pGEM target in the presence of HIV-1 IN and LEDGF (lane 1), IN alone (lane 2), or in the absence of both proteins (lane 3) were separated in 0.6% agarose along with 1-kb ladder DNA (lane L) (top gel). A lane with a sample identical to that in lane 1 was excised from the gel. In this gel slice, two wells were created for parallel separation of the 1-kb DNA ladder and another aliquot of sample 1. The DNA was then separated in the perpendicular dir [file ppat.1000259.s005.tif]
